# A comprehensive review of artificial intelligence for pharmacology research

**DOI:** 10.3389/fgene.2024.1450529

**Published:** 2024-09-03

**Authors:** Bing Li, Kan Tan, Angelyn R. Lao, Haiying Wang, Huiru Zheng, Le Zhang

**Affiliations:** ^1^ College of Computer Science, Sichuan University, Chengdu, China; ^2^ Department of Mathematics and Statistics, De La Salle University, Manila, Philippines; ^3^ School of Computing, Ulster University, Belfast, United Kingdom

**Keywords:** artificial intelligence, pharmacology, drug discovery, compound pharmacokinetic prediction, clinical pharmacology

## Abstract

With the innovation and advancement of artificial intelligence, more and more artificial intelligence techniques are employed in drug research, biomedical frontier research, and clinical medicine practice, especially, in the field of pharmacology research. Thus, this review focuses on the applications of artificial intelligence in drug discovery, compound pharmacokinetic prediction, and clinical pharmacology. We briefly introduced the basic knowledge and development of artificial intelligence, presented a comprehensive review, and then summarized the latest studies and discussed the strengths and limitations of artificial intelligence models. Additionally, we highlighted several important studies and pointed out possible research directions.

## 1 Introduction

**FIGURE 1:**
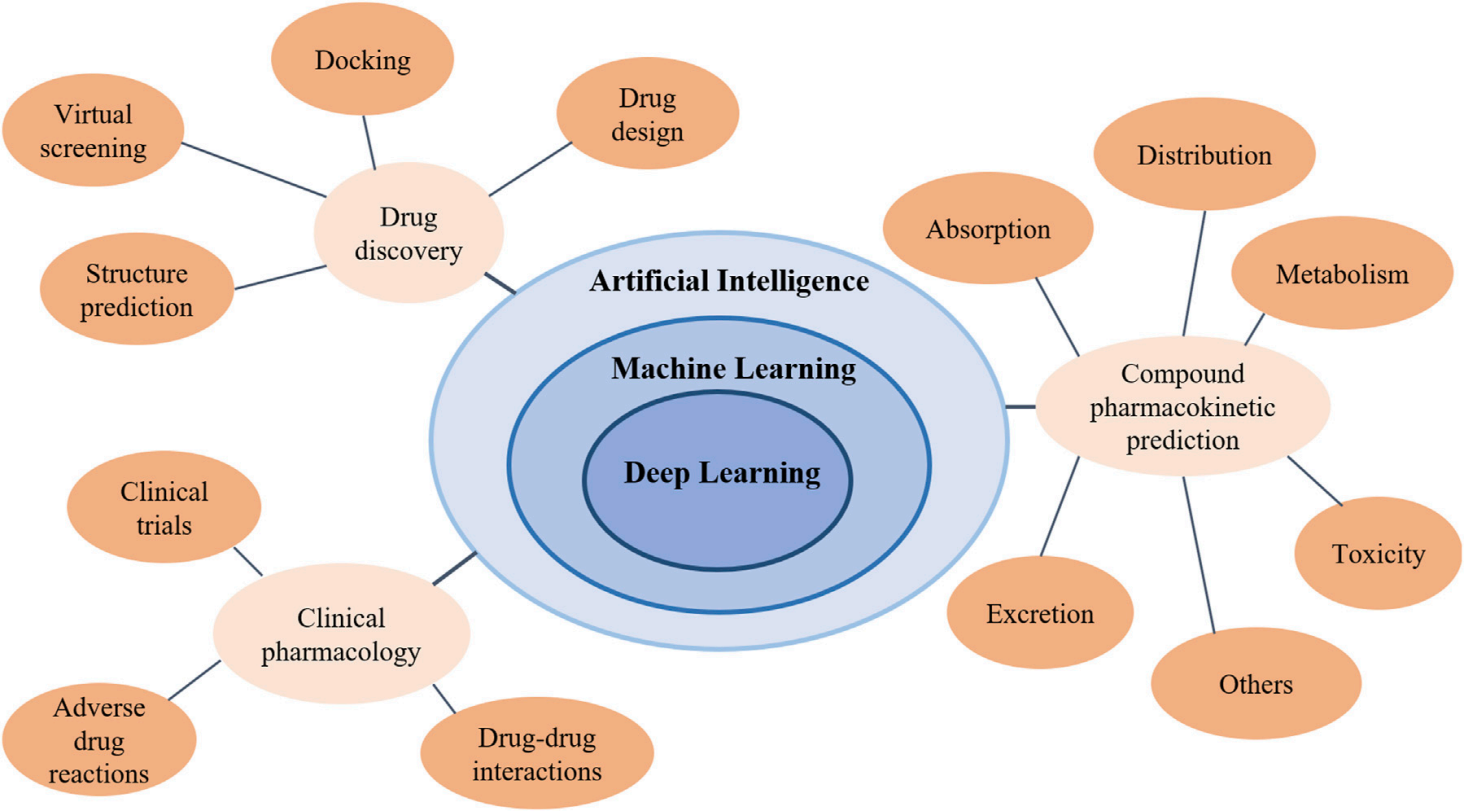
The relationship between artificial intelligence, machine learning, and deep learning and the applications of artificial intelligence in pharmacology research.

Artificial intelligence (AI) is defined as the intelligence exhibited by artificial entities to solve complex problems, and is generally considered to be a system of computers or machines (Kumar et al., 2012). With the emergence of big data and the improvement of computing power, machine learning, artificial neural networks, and deep learning (Gao et al., 2022; Song et al., 2022; Gao et al., 2023) have been developing rapidly and continued to integrate other disciplines in recent years, achieving great success in theory and application (Chaturvedula et al., 2019; Brown et al., 2020; Woschank et al., 2020; Alzubaidi et al., 2021; Mohsen et al., 2023). Figure 1 shows the relationship between AI and related concepts such as machine learning, artificial intelligence, and deep learning. Meanwhile, Figure 1 shows the applications of artificial intelligence in pharmacology research.

**FIGURE 2:**
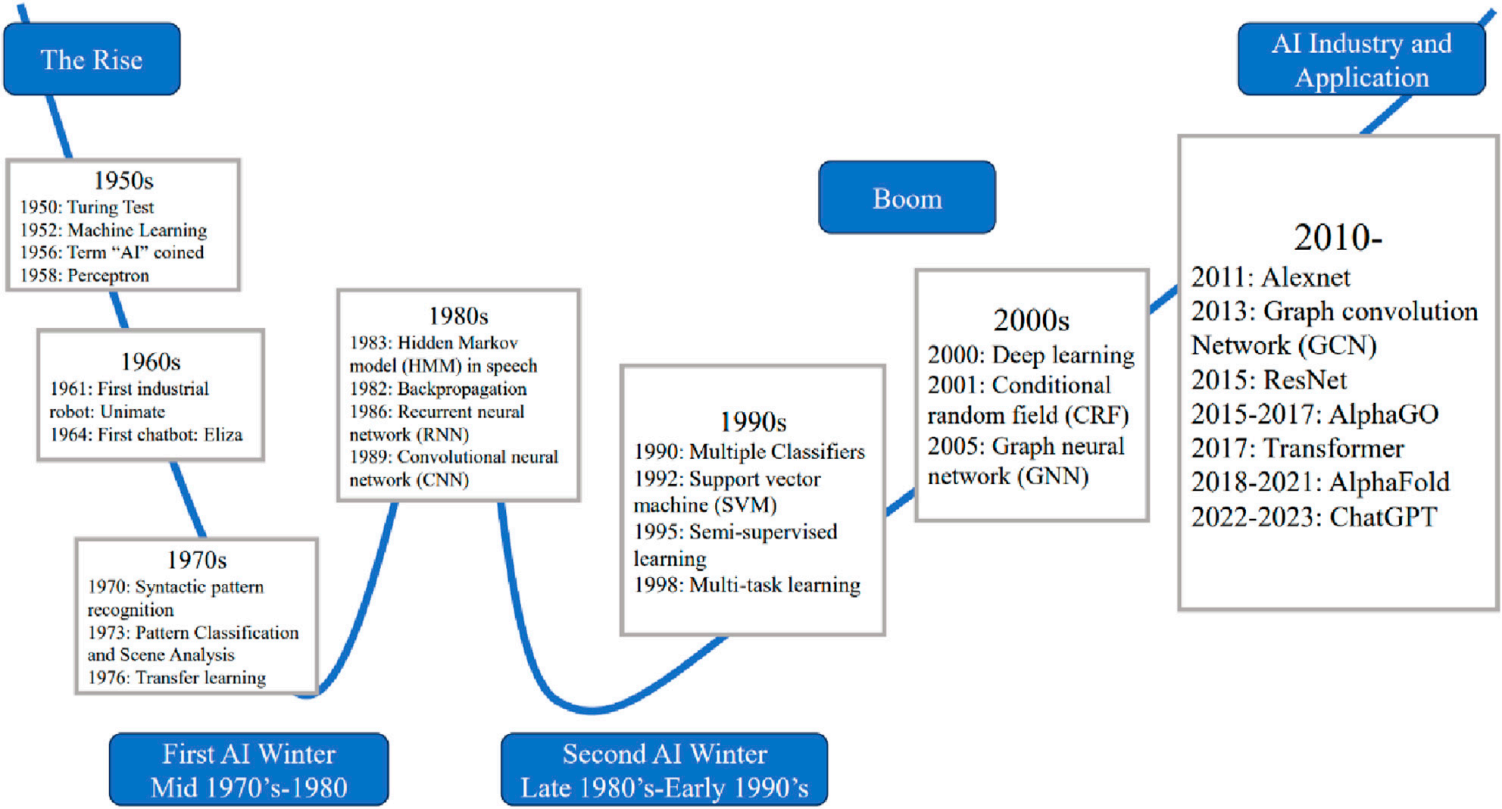
Timeline of the development and application of artificial intelligence.

The development of AI can be traced back to the 1940s, and its historical process and development have been detailed in many previous reviews (Muggleton, 2014; Haenlein and Kaplan, 2019). In recent decades, the widespread application of neural networks, such as convolutional neural networks (CNNs), recurrent neural networks (RNNs), graph neural networks (GNNs) and deep neural networks (DNNs) (Gao et al., 2021; Lai et al., 2022), as well as the development of deep learning algorithms, such as ResNet (He et al., 2015; Zhang et al., 2024), Attention and Transformer (Vaswani et al., 2017; You et al., 2022b), have driven the development of neural networks and deep learning, and further optimized the application performance of AI algorithms in various fields (Alzubaidi et al., 2021). Figure 2 briefly extracts and exhibits the most important algorithms proposed during the development of AI.

The study of pharmacology originated in the mid-19th century and it covers a very wide range of fields (Vallance and Smart, 2006). The driving force of pharmacology is to understand and quantify the effects of drugs on physiology, including exploring the action of drugs, the mechanism of action of drugs, and the active ingredients of drugs (Vallance and Smart, 2006). It is generally believed that the scope of pharmacology (Vallance and Smart, 2006) is comprised of drug discovery, design, explanation of mechanisms, drug metabolism and actual clinical research, etc., Therefore, pharmacology is a very complex and comprehensive science.

The contribution of AI in pharmacology research does not appear suddenly, but with the development of AI and pharmacology themselves, mutual promotion and growth. Research on the combination of AI and pharmacology has been proposed for a long time (So and Karplus, 1996a; b). It is worth noting that although methods such as neural networks were proposed for use in QSAR (Quantitative Structure-activity Relationship) models at that time (So and Karplus, 1996a), there are at least some difference or progress for now, namely more abundant and suitable AI models for different situations as mentioned above, more standard data sets and research community building (Su et al., 2019), more various kind of descriptors and wider applications in pharmacology as summarized below.

Pharmacology is a very complex study involving a lot of computing, data statistics and analysis. A number of AI methods (Zhang et al., 2019b; Zhang et al., 2021c; Zhang et al., 2021d; You et al., 2022a; Zhang et al., 2023b) have been used in pharmacology research, where the most widely used fields are AI-assisted drug discovery and design (Paul et al., 2021), prediction of compound pharmacokinetics (Obrezanova, 2023) and clinical pharmacology (Johnson et al., 2023). Thus, this review will focus on the application of AI in these three areas (shown in Figure 1), and introduce latest research methods and models in the following sections.

## 2 AI-assisted drug discovery and design

Classical molecular drug discovery and design encounters several problems and challenges such as long development time, low clinical success rate and high cost. In general, it takes about 13.5 years for a drug molecule to be developed and approved for marketing, and the total cost to develop a new drug is about $2.6 billion (DiMasi et al., 2016). Moreover, it becomes more difficult to develop a novel clinical drug due to these costs rising every year (DiMasi et al., 2016).

Recently, the development and application of AI has facilitated the research related to drug discovery and drug design, which is reflected in three main aspects: 1. Using AI to predict the structure of proteins and RNA; 2. AI-assisted drug discovery, and 3. Using AI for drug design.

### 2.1 Using AI to predict the structure of proteins and RNA

The analysis and investigation of the 3D structure of proteins and the related molecules is the precursor for drug discovery and design. It is highly accurate to obtain the 3D structure of proteins and RNA by physical and chemical experimental methods, but it requires a lot of manpower and financial resources. Therefore, recent studies employ computing techniques to predict the 3D structure of molecules (Huang B. et al., 2023).

Classical 3D structure prediction methods consist of *de novo* modeling, fragment assembly, and homology modeling, the mechanism of which are based on rule-based computing and splicing but not using AI for 3D structure prediction (Li et al., 2020; Huang B. et al., 2023). Thus, before AlphaFold was innovated, the application of AI in structure prediction focused more on the prediction of features related to primary and secondary structures rather than very complicated 3D structures (Kuhlman and Bradley, 2019).

With the release of AlphaFold by DeepMind (Jumper et al., 2021) and RoseTTAFold by David Baker’s team (Baek et al., 2021), scientists proposed many novel ideas for 3D structure prediction of proteins and molecules. A comparison that may be inappropriate but illustrates the significance is: Tunyasuvunakool et al. (Tunyasuvunakool et al., 2021) successfully predicted 98.5% of human proteins by AlphaFold, and 58% of the residues had confident prediction results and 36% of all residues predictions had very high confidence. In contrast, decades of human structural experiments have only determined 17% of all residues.

Moreover, Zhang et al. (2023f) investigated the virtual screening performance for 37 common drug targets, which have AlphaFold2 predicted structures and experimental structures. The AlphaFold2 predicted structures show similar performance with experimental structures in early enrichment in a subset of 27 targets. It demonstrates that AlphaFold2 structures have great potential in virtual screening after proper preparation and refinement.

For more details on the impact and changes AlphaFold has brought to the field of structural biology, Yang et al. (2023) summarized the related studies and applications in structural biology, drug discovery, protein design and so on, and then they considered that AlphaFold has achieved great success and significantly remodeled structural biology (Bertoline et al., 2023; Yang et al., 2023).

Despite the great success, AlphaFold has many limitations. Besides static structures, it is very important for us to study and predict the structural dynamics of unstructured molecules, such as allosteric drugs and their active state, which is the conformational ensemble (Fisher and Stultz, 2011; Nussinov et al., 2023). However, AlphaFold and the related AI methods currently do not provide such solutions.

There is no doubt that the emergence of AlphaFold has brought great changes to the study of protein structure. As for now, an optimized AlphaFold predicted structure can provide a reasonable starting point for physical-based molecular dynamics simulations, making them more effective in drug discovery (Gomes et al., 2022; Schauperl and Denny, 2022; Nussinov et al., 2023). However, there are still many limitations remaining to be solved and optimized. For example, how to further advance and optimize AI methods to predict structure and conformational ensemble for protein complexes and unstructured proteins should be the most important research direction in the future.

### 2.2 AI-assisted drug discovery

From the random screening and empirical observation of the effects of natural products on disease to discover drugs, to the use of high-throughput screening (HTS) to batch screen drugs against molecular targets (Macarron et al., 2011), and to computer aided drug design (CADD) (Yu and MacKerell, 2017), the approach to discover novel drugs continues to be revolutionized.

With the rapid development of the computational power and algorithms of AI, as well as the rapid expansion of drug-like available chemical space, a new revolution is coming for drug discovery (Maia et al., 2020; Sadybekov and Katritch, 2023). Since computer-aided drug discovery not only can decrease the drug development cycle, but also it can reduce the cost of the clinical trial phase, related studies were carried out to assist and accelerate drug discovery, which include the development of virtual screening (Lavecchia and Di Giovanni, 2013), molecular dynamic simulation (Durrant and McCammon, 2011) and molecular docking (Meng et al., 2011; Fan et al., 2019; Thafar et al., 2019).

In these studies, computer-aided drug development has been categorized into two main approaches according to whether the molecular structure is known or not. One is the structure-based approach and the other is the ligand-based approach. Ligand-based approaches use similarities of known active molecules to carry out modeling and computing, whereas structure-based approaches focus on computing and prediction for binding affinity. Next, we will detail the application of AI methods for these two approaches (Yu and MacKerell, 2017; Yang et al., 2019; Maia et al., 2020; Paul et al., 2021).

For ligand-based approaches, similar to traditional ligand-based QSAR methods, many researchers build up QSAR models (Neves et al., 2018) to realize ligand-based virtual screening by using artificial intelligence methods (Lima et al., 2016; Dai and Guo, 2019). Additionally, compared with traditional machine learning methods, neural networks and other algorithms are used (as shown int Table 1). For example, DNNs were employed to predict QSAR models to screen new dipeptidyl peptidase-4 (DPP-4) inhibitors for the treatment of diabetes mellitus type 2 (Bustamam et al., 2021). Also, DNNs (Xiao et al., 2018) and various AI-driven ligand-based virtual screening tools and platforms have been developed and used (Amendola and Cosconati, 2021; Oliveira et al., 2023).

**TABLE 1:** Table summaries for some ligand-based models.

Models	Feature	Targets	Benchmark methods	Citation
DNN	2D	EGFR, SRC, mTOR, PIK3CA, MMP1 and MMP2	RF	Xiao et al. (2018)
LBS	2D	Inhibitors of Rho kinase 2 and HIV-1 integrase multimerization	KNN, SVM	Dai and Guo (2019)
DNN	2D	Dipeptidyl peptidase-4 (DPP-4) inhibitors	PCA, SPCA	Bustamam et al. (2021)
RMD	2D	DUD-E and MUV	RF, GB, LR, NB	Amendola and Cosconati (2021)
DNN	2D	ZINC15	—	Gentile et al. (2022)

Abbreviations: LBS, local beta screening; KNN, K-nearest neighbors; SVM, support vector machine; DNN, deep neural network; PCA, principal component analysis; SPCA, sparse principal component analysis; RMD, random matrix discriminant; RF, random forest; GB, gradient boosting; LR, logistic regression; NB, naïve bayes.

**FIGURE 3:**
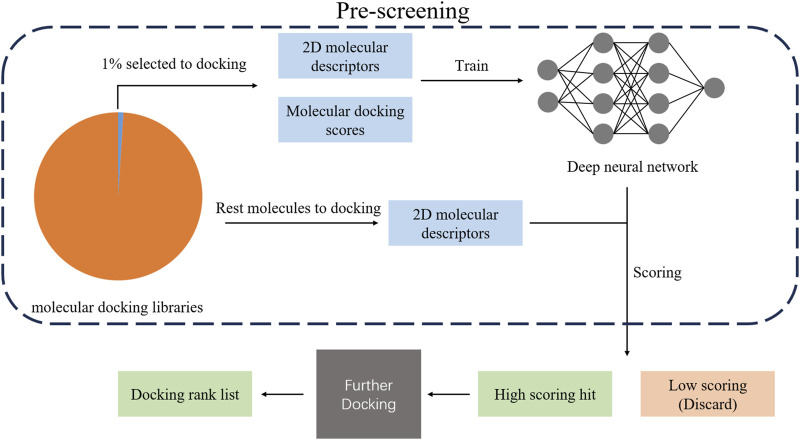
Illustration for pre-screening DNN model.

In addition to the above approaches, a recent study proposed a deep learning-based deep docking platform (shown in Figure 3), which can train a DNN model by employing a portion of selected data from a huge number of molecular docking libraries. The DNN model is used to predict the docking scores for optional 2-dimensional molecular descriptors and candidate molecules from the molecular docking libraries. According to the predicted score, top-scoring candidate molecules will be selected to carry out further docking (models with higher accuracy), and low-scoring molecules will be filtered out. Since the computing load of virtual screening can be decreased by using a DNN for pre-screening, it provides a novel idea to explore the high-dimensional chemical space efficiently (Gentile et al., 2022).

For structure-based approaches, a hot study direction is to propose the binding affinity models [binding affinity scoring functions (Meli et al., 2022; Sadybekov and Katritch, 2023)] from the known ligand activities and corresponding protein-ligand 3D structural data.

The scoring functions are categorized into the following four types (Meli et al., 2022): Physics-Based (Force-Field Based) scoring function, Empirical (Regression-Based) scoring function, Knowledge-Based (Potential-Based) scoring function, and Descriptor-based or Machine Learning-Based scoring function.

Descriptor-based and traditional machine learning scoring functions have been proposed and used since the 1990s, which are usually based on SVM, random forests, and gradient boosting (Meli et al., 2022). They are often used to explore the nonlinear relationship between descriptors and binding affinities (Meli et al., 2022). With the development of neural networks and deep learning, scientists have proposed many binding affinity models based on feed-forward neural networks (Ashtawy and Mahapatra, 2015; Meli et al., 2021), convolutional neural networks (Jiménez et al., 2018; Stepniewska-Dziubinska et al., 2020), graph neural networks (Gaudelet et al., 2021; Son and Kim, 2021), and other neural networks (Ashtawy and Mahapatra, 2018; Jones et al., 2021).

Both descriptors and models are the key factors for binding affinity prediction, and impact the final prediction ability. Table 2 lists some researches with different descriptors and models, from which it can be found that descriptors with strong expression ability together with appropriate and powerful models, make up good prediction models.

**TABLE 2:** Performance comparison for structure-based models.

Models	Algorithm	Descriptor	$R_{p}$	RMSE	Citation
RFScore-V3	RF	intermolecular contacts and Autodock Vina features	0.800	1.390	Li et al. (2015)
AGL-Score	GBT	algebraic graph descriptor	0.833	1.271	Nguyen and Wei (2019)
KDeep	CNN	3D voxel representation	0.820	1.270	Jiménez et al. (2018)
PLEC	NN	PLEC FP	0.817	—	Wójcikowski et al. (2019)
AEVs	NN	atomic environment vectors	0.83	1.22	Meli et al. (2021)
IGN	GNN	molecular graph	0.837	1.220	Jiang et al. (2021)
GraphscoreDTA	GNN	molecular graph	0.831	1.249	Wang et al. (2023)

Abbreviations: GNN, graph neural network; NN, neural network; CNN, convolutional neural network; RF, random forest; GBT, gradient boosting tree.

For descriptors, more detailed and accurate description for protein-ligand interactions could lead to the improvement of prediction ability. For example, Wójcikowski et al. (2019) presented a Protein-Ligand Extended Connectivity (PLEC) Fingerprint to encode protein-ligand interactions and build up different models to predict protein–ligand affinities, including linear regression, random forest and neural network. The Pearson correlation coefficient obtained on the CASF-2016 benchmark is 0.817. Meli et al. (2021) proposed to employ atomic environment vectors (AEVs) and feed-forward neural networks to predict protein-ligand binding affinity, which achieved RMSE of 1.22 and Pearson’s correlation coefficient of 0.83 on the CASF-2016 benchmark. Both researches focus on the enrichment of descriptors, suggesting a research direction, but whether it works remains to be discussed, which will be mentioned later.

Besides the descriptors study, it is very important to build up a suitable and powerful model. Wang et al. (2023) developed GraphscoreDTA, which adopts Vina distance optimization by combining graph neural network, bitransport information mechanism and physics-based distance terms. GraphscoreDTA model obtained RMSE of 1.249 and Pearson’s correlation coefficient of 0.831 on the CASF-2016 benchmark. Jiang et al. (2021) proposed InteractionGraphNet (IGN), stacked by two independent graph convolution modules, which are trained to learn intramolecular and intermolecular interactions. IGN model obtained the RMSE of 1.220 and Pearson’s correlation coefficient of 0.837 on the CASF-2016 benchmark in the best case.

For descriptors, despite numbers of descriptors for protein and ligand presentation are proposed and discussed as mentioned above, Volkov et al. indicated (Volkov et al., 2022) that providing more docking details, such as an explicit description of protein-ligand noncovalent interactions, cannot demonstrate an explicit advantage when training neural network models rather than using only ligand or protein descriptors. Especially, memory largely dominates the learning process of deep neural networks in most cases. Thus, it will become a meaningful research direction to investigate how to represent the structures of ligands and proteins, and how to use optimal descriptors to represent ligands and proteins (Meli et al., 2022; Gu et al., 2023) rather than capturing information about their binding. After that, choosing the suitable models to make better use of the information provided by descriptors will also be an important part of affecting the ability of the model.

Moreover, it is worth noting that the Pearson correlation coefficient is used to evaluate the binding affinity prediction ability of the scoring function, while RMSE is used to evaluate the docking ability of the scoring function, which is, the ability to select the native binding conformation of the ligand from a series of poses (Vittorio et al., 2024). In molecular docking and virtual screening practice, pose prediction and affinity prediction are two complementary tasks. Better molecular docking results can be obtained by constantly adjusting pose and calculating its binding affinity (Meli et al., 2022). Previous study shows that binding affinity prediction ability and docking ability are not closely correlated for many existing scoring functions (Vittorio et al., 2024). Predictions for binding affinity are mostly based on single binding conformation of the ligand found in the experimental complex, which may be partly to blame for the underperformance of these scoring functions in actual virtual screening tasks (Gabel et al., 2014; Shen et al., 2021), and training the scoring functions using the structure of docking pose (Francoeur et al., 2020), or the application of Data Set Augmentation techniques (Scantlebury et al., 2020), may help to improve the robustness of those scoring functions. In addition, most of the current databases provide only well-bound protein and ligand data, i.e., positive data, but lack suboptimal binding affinity, i.e., negative data. Therefore, it will become a hot research direction (David et al., 2020; Xu et al., 2020; Kimber et al., 2021; Sadybekov and Katritch, 2023) to collect and provide these data to improve the performance for AI models.

### 2.3 Using AI for drug design

Strictly speaking, drug discovery is to discover potential drugs by computational, experimental, and clinical models, whereas drug design is to design and develop new drugs based on known signaling pathways and biological targets, i.e., designing molecules that match their target molecules in shape and charge (Zhou and Zhong, 2017). Here, we will focus on the applications of AI in drug design, namely *de novo* drug design (Wang et al., 2022).

*De novo* drug design (Mouchlis et al., 2021) refers to generating a series of new molecules that meet certain constraints by developing generative algorithms. The advantage of this approach is that we can design a drug in such a greater chemical space that could develop more targeted drugs for the treatment of diseases. However, it encounters such a challenge that is how to generate a new molecule, which is stable and easy to produce without a starting template. Traditional *de novo* drug design is comprised of structure-based, ligand-based, sampling-based, and evolutionary algorithm-based approaches, which are detailed by Mouchlis et al. (2021) due to space limitations.

Generally, there are four basic types of models to do *de novo* drug design, which are RNN-based model, Autoencoder-based model (AE, also known as encoder-decoder model), GAN-based model (Generative Adversarial Network), and reinforcement learning-based model. In practice, most algorithms are based on one or a combination of these four structures (Wang et al., 2022).

RNN related models (Li et al., 2018; Kotsias et al., 2020; Moret et al., 2020; van Deursen et al., 2020) generate new molecules with the highest probability by taking the output of the previous layer as input, and iterate to continuously optimize its output molecules.

For example, Urbina et al. (2022) recently proposed MegaSyn, which is a tool integrating generative molecular design and automated analog design into synthetic viability prediction. MegaSyn employed SMILES-based RNN generative model and its performance is demonstrated by several case studies (Urbina et al., 2022).

In addition, several studies combine RNN and reinforcement learning (Popova et al., 2018; Liu et al., 2019; Ståhl et al., 2019; Blaschke et al., 2020) to construct *de novo* drug design models. For example, Hu et al. (2023) proposed a *de novo* drug design model based on Stack-RNN, multi-objective reward-weighted sum and reinforcement learning. By multi-objective reward-weighted sum, it solved the potential conflicts between different properties of the generated molecules. Moreover, since it is a multi-objective optimization task, it also prevents the generated molecules to be extremely biased towards a certain property. Their model achieved a validity of 97.3%, an internal diversity of 0.8613, and increased desirable molecules from 55.9% to 92%.

Autoencoder is an unsupervised learning model consisting of the encoder and decoder (Gómez-Bombarelli et al., 2018). The encoder converts the input molecules into vectors in the latent space, and the decoder can revert the vectors into molecular representations. Therefore, we can adjust the molecular design by changing the vectors in the latent space. Variational autoencoder (VAE) is the first AE framework for molecule design (Gómez-Bombarelli et al., 2018). Several studies have subsequently made improvements and enhancements based on this framework (Kusner et al., 2017; Skalic et al., 2019; Ye et al., 2021). For example, Lim et al. (2018) introduced several molecular properties into the latent space to carry out conditional control and adjustment for the generated molecules. Liu et al. (2018) introduced graph into variational autoencoder, where both encoder and decoder are graph structured. Moreover, deep generative model was introduced by Samanta et al. (2019), which can effectively discover plausible, diverse and novel molecules and generate molecules that maximize the property of interest.

Furthermore, adversarial autoencoder (AAE) algorithms, which is the combination of VAE and GAN, can generate target-specific molecules (Kadurin et al., 2017; Polykovskiy et al., 2018; Prykhodko et al., 2019). For example, Prykhodko et al. (2019) proposed a deep learning architecture LatentGAN, which is able to generate both drug-like compounds and target-biased compounds. A GAN is trained to generate fake latent vector which is taken as the input for the decoder in VAE and then generates new molecules for their model. Besides the above models, some other studies based on AE include Heteroencoder (Bjerrum and Sattarov, 2018), GTM-RNN (Sattarov et al., 2019) and reinforcement learning based GENTRL (Zhavoronkov et al., 2019).

GAN (Aggarwal et al., 2021) consists of a generator and a discriminator, where the generator generates new molecules and the discriminator distinguish whether the input molecules are real or generated by the generator. Performance of the generator and the discriminator can be improved by continuously training. And in practice, GAN is often used together with other models.

Reinforcement learning (Nian et al., 2020; Pereira et al., 2021; Atance et al., 2022; Korshunova et al., 2022; Fang et al., 2023) consists of a generative model and a drug design agent model. Generative model is generally constructed by a multi-layer neural network, which generates a new state as an output based on the results of the previous generation or the initial state by the neural network. The outputs are evaluated by the drug agent model, enabling iterations to optimize the designed molecule. Reinforcement learning is not only always combined with other generative algorithms like RNN mentioned above, but also works with GAN, such as ORGAN (Guimaraes et al., 2017), ORGANIC (Sanchez-Lengeling et al., 2017) and ATNC (Putin et al., 2018) to construct *de novo* drug design models. Especially, Abbasi et al. (2022) proposed a framework comprising Encoder–Decoder architecture, Wasserstein GAN with gradient penalty and optimization step based on Feedback GAN (Pereira et al., 2021), which can be regarded as the combination of autoencoder, GAN and reinforcement learning. Their Encoder–Decoder model correctly reconstructed 99% of the datasets, including stereochemical information, and generated compounds with 62.3% validity, 0.88 internal diversity and 0.94 external diversity.

The applications of AI for *de novo* drug design are still in its beginning stages, and their performance have not significantly surpassed that of traditional and evolutionary algorithm-based models (Wang et al., 2022; Zhang et al., 2023e). We still lack a comprehensive target-specific *de novo* drug design platform, while a large amount of work is currently on theoretical studies for the development of new algorithms (Bai et al., 2021; Wang et al., 2022).

Additionally, synthetic feasibility is still an important question without enough concerns. A reachable solution is to provide synthesizability scoring, like synthetic accessibility (SA) score (Ertl and Schuffenhauer, 2009) and synthetic complexity (SC) score (Coley et al., 2018), which can easily compute scores of syntheses for a target molecule and exclude unsynthesizable molecules. Besides synthesizability scoring functions, there are also ways to make sure the synthetic feasibility, like synthesis planning, synthesis prediction and Fragment/synthesis-driven molecular construction and generative models, which are detailed in this review (Stanley and Segler, 2023).

In conclusion, AI shows great potential in *de novo* drug design, but its research is still in its infancy. A great deal of research on algorithmic exploration and practical application is yet to be explored further in depth.

## 3 Artificial intelligence for compound pharmacokinetics prediction

In scenarios such as drug development, drug design, and drug dosage exploration, the pharmacokinetic studies of candidate compounds, i.e., the studies of properties like drug absorption, distribution, metabolism, excretion, and toxicity (ADMET), are essential, because any drug candidate must be tested for ADMET properties to guarantee the effectiveness and safety of the drug (Tsaioun et al., 2009).

Therefore, we can significantly reduce the chemical searching space, increase the success rate of drug development, and decrease its cost (Tran et al., 2023c) by employing AI technology to build up predictive models for pharmacokinetics, validate ADMET properties for drug candidates in the early stages of drug development, and screen out the undesired drugs, When predicting ADMET and physicochemical properties, each process is corresponding to a number of important features, including but not limited to those shown in Table 3 (Dulsat et al., 2023). Both traditional machine learning and neural network methods received good predictive effect using these features. Due to the limitation of space, the development process and detailed studies can be found in these reviews (Chandrasekaran et al., 2018; Yang et al., 2019; Danishuddin et al., 2022; Dulsat et al., 2023; Tran et al., 2023a; Tran et al., 2023c; Tran et al., 2023b), and the following highlighted several important studies and recent advances of AI in ADMET prediction.

**TABLE 3:** Important features for ADMET and Physicochemical properties.

	Important features
Absorption	Human intestinal absorption (HIA), Human oral bioavailability (HOB, F%), P-Glycoprotein inhibitor/substrate, Caco-2/MDCK permeability
Distribution	plasma protein binding (PPB), fraction unbound in plasma (Fu), blood–brain barrier (BBB), volume of distribution (Vd)
Metabolism	Cytochrome P450 isoforms (CYP450s) inhibitor/substrate, Human liver microsomes (HLM), Metabolites and Sites
Excretion	Clearance (Cl), Half-Life ( $T_{\frac{1}{2}}$ )
Toxicity	Acute toxicity, Carcinogenicity, human ether-a-go-go-related gene (hERG), and Ames test
Physicochemical Properties	Lipophilicity (log P), Aqueous Solubility (log S), Acid dissociation constant ( ${pK}_{a}$ )

Since previous machine learning studies (Aleksić et al., 2022) indicated that choosing different traditional machine learning models and increasing the amount of training data cannot significantly affect the prediction accuracy, it implied that limited improvement can be achieved by using machine learning methods. Therefore, many studies have started to use various neural network models to predict pharmacokinetic parameters as described below.

For example, DNN (Sakiyama et al., 2021; Kumar et al., 2022; Mazumdar et al., 2023), RNN (Alsenan et al., 2020) and CNN (Alsenan et al., 2021) are used to predict blood-brain barrier permeability. DNN is used to predict CYP450s inhibition (Park et al., 2022). Multi-task CNN is used to predict *in vitro* clearance from molecular images (Martínez Mora et al., 2022). GCN (Chen et al., 2021) and multi-task DNN (Sharma et al., 2023) are used to predict toxicity. These studies show that modelling with neural networks is commonly used to predict the ADMET and physicochemical properties of compounds.

More importantly, unlike independently constructed models that predict single or partial properties of ADMET, recent studies (Dulsat et al., 2023) can predict multiple important features of ADMET and physicochemical properties by integrating multiple models. Representative works include ADMETlab (Dong et al., 2018), ADMETlab 2.0 (Xiong et al., 2021), admetSAR (Cheng et al., 2012), admetSAR 2.0 (Ye et al., 2019), FAF-Drugs4 (Lagorce et al., 2017), FP-ADMET (Venkatraman, 2021), Interpretable-ADMET (Wei et al., 2022), and HelixADMET (Zhang et al., 2022).

In these works, ADMETlab (Dong et al., 2018) can predict a wide range of coverage with good accuracy and precision (Dulsat et al., 2023), which has 31 ADMET endpoints prediction in ADMETlab Version 1.0, and increases to 88 in ADMETlab Version 2.0. Furthermore, ADMETlab Version 2.0 (Xiong et al., 2021) increases the quality and quantity of data for model construction.

In terms of modeling methods, ADMETlab Version 1.0 uses traditional machine learning algorithms, including random forest (RF) (Cao et al., 2012), support vector machine (SVM) (Cao et al., 2015), recursive partition regression (RP) (Strobl et al., 2009), partial least squares (PLS) (Cao et al., 2010), naïve Bayes (NB) (Jiang et al., 2018), and decision tree (DT) (Xia et al., 2018), to build QSAR regression models and classification models for ADMET properties. ADMETlab Version 2.0 employs attention mechanism and graph convolutional neural network to simultaneously learn the regression and classification tasks in ADMET prediction. And it proposes a multi-task graph attention (MGA) framework, where different attention layers can be generated for various tasks to generate specific feature maps (customized fingerprints).

Compared with Version 1.0, ADMETlab Version 2.0 not only increases its precision and accuracy, but also improves computational efficiency by employing graphs to represent molecules instead of the traditional descriptor-based representation. Table 4 shows the comparisons between ADMETlab Version 1.0 and Version 2.0, which is a portrayal of the comparison between neural network model and traditional machine learning methods. More detailed comparisons and evaluations between different works can be found in this review (Dulsat et al., 2023).

**TABLE 4:** Comparisons between ADMETlab Version 1.0 and Version 2.0

Regression	ADMETlab version 1.0			ADMETlab version 2.0		
	Model	R2	RMSE	Model	R2	RMSE
LogS	RF	0.979	0.712	MGA	0.854	0.850
LogD7.4	RF	0.874	0.605	MGA	0.892	0.462
Caco-2	RF	0.824	0.290	MGA	0.746	0.307
PPB	RF	0.682	18.044	MGA	0.733	0.135
VD	RF	0.556	0.948	MGA	0.782	0.670

Classification	ADMETlab Version 1.0			ADMETlab Version 2.0		
	model	AUC	ACC	model	AUC	ACC
HIA	RF	0.846	0.782	MGA	0.866	0.924
$F_{20\%}$	RF	0.759	0.689	MGA	0.833	0.75
$F_{30\%}$	RF	0.715	0.669	MGA	0.848	0.802
BBB	SVM	0.948	0.926	MGA	0.908	0.862
Pgp-inhibitor	SVM	0.908	0.848	MGA	0.922	0.867
Pgp-substrate	SVM	0.899	0.824	MGA	0.84	0.768
CYP1A2-inhibitor	SVM	0.928	0.849	MGA	0.928	0.852
CYP1A2-substrate	RF	0.801	0.702	MGA	0.737	0.649
CYP3A4-inhibitor	SVM	0.901	0.817	MGA	0.921	0.832
CYP3A4-substrate	RF	0.835	0.757	MGA	0.776	0.713
CYP2C19-inhibitor	SVM	0.893	0.822	MGA	0.913	0.839
CYP2C19-substrate	RF	0.816	0.74	MGA	0.758	0.654
CYP2C9-inhibitor	SVM	0.9	0.837	MGA	0.919	0.841
CYP2C9-substrate	RF	0.819	0.728	MGA	0.725	0.707
CYP2D6-inhibitor	RF	0.868	0.793	MGA	0.892	0.824
CYP2D6-substrate	RF	0.823	0.748	MGA	0.847	0.775
hERG	RF	0.879	0.844	MGA	0.943	0.889
H-HT	RF	0.71	0.689	MGA	0.814	0.72
Ames	RF	0.89	0.82	MGA	0.902	0.807
Skin Sensitization	RF	0.76	0.706	MGA	0.707	0.775
DILI	RF	0.904	0.84	MGA	0.924	0.894
FDAMDD	RF	0.904	0.832	MGA	0.804	0.736

Abbreviations: RF, random forest; SVM, support vector machine; MGA, Multi-task graph attention.

ADMETlab Version 1.0 took more than 2 hours while ADMETlab Version 2.0 only took 84 s in the computational test for 1,000 molecules, since ADMETlab Version 2.0 improved the performance of regression and classification for many properties (shown in Table 4). For examples, the $R^{2}$ of Log D7.4 increased from 0.874 to 0.892, the $R^{2}$ of PPB increased from 0.682 to 0.733, and the $R^{2}$ of VD increased from 0.556 to 0.782. In the classification task, the AUC of HIA increased from 0.831 to 0.866, and the AUC of hERG increased from 0.873 to 0.943. However, the performance of several properties was decreased, such as BBB, Pgp-substrate, Log S, Caco-2 and so on, which may be due to the use of different datasets. Besides the above, several properties, which are assessed ambiguously in ADMETlab Version 1.0 due to the limitation of algorithms, like CL and Half-Life, are well predicted and evaluated in ADMETlab Version 2.0. And then, the regression task of ADMETlab Version 2.0 for CL obtained $R^{2}$ of 0.678 and RMSE of 3.375. The classification task for Half-Life obtained AUC of 0.801 and accuracy of 0.727 for ADMETlab Version 2.0.

Similar to the modeling for ADMETlab Version 2.0, Interpretable-ADMET uses graph convolutional neural networks and graph attention networks (Wei et al., 2022), and HelixADMET is based on graph neural networks (Zhang et al., 2022). The above methods have made great progress in ADMET prediction, which have also been compared and evaluated by Dulsat et al. (Dulsat et al., 2023) in detail. Since these works employ graph presentation and graph neural networks, it suggests feasible directions for subsequent studies on ADMET prediction for both molecular expression and model selection. The wide use of these ADMET prediction tools also demonstrates the great potential for deep learning and graph neural networks in ADMET prediction.

## 4 Artificial intelligence for clinical pharmacology

Besides the above work related to drug discovery, drug design and pharmacokinetics prediction, AI has many applications in clinical pharmacology, such as using AI to optimize clinical trial design, simulate clinical trial results, optimize drug treatment process, predict drug interactions and adverse reactions, and so on.

### 4.1 AI in clinical trials

Clinical trial is an important stage in the development of drugs. The failure during clinical trials will result in a huge loss of time and cost. Thus, using AI to assist clinical trials will effectively improve efficiency and success rate (Askin et al., 2023).

As we know, it is one of the most challenging steps to recruit the relevant patients during the clinical trial design. For this reason, we usually employ machine learning algorithms to screen the patients, match them to the trial’s inclusion criteria through multiple aspects of data, and guarantee that the included patients are suitable for that clinical trial (Harrer et al., 2019; Beck et al., 2020; Beaulieu et al., 2021; Vazquez et al., 2021; Weissler et al., 2021).

Also, AI can be used to predict and select these patients who will progress and reach the endpoint more quickly. And then, the duration of drug trials can be potentially reduced (Lee and Lee, 2020). When the trial is in progress, AI can predictively determine participants who may drop out midway through the electronic medical record, and to improve the completion rate of the trial (Krittanawong et al., 2019) by reminding the experimentalist to pay extra attention to these participants.

More notably, with the development of large language models, AI becomes increasingly capable of simulating human-like responses and behaviors in social science research, to the point where AI can be used to complete certain trials instead of humans (Grossmann et al., 2023). Like oncology drug research, AI algorithms can predict drugs’ performance in clinical trials.

One of the previous studies (Kolla et al., 2021) used Causal AI to build *in silico* trials, which employed clinical data to construct simulated cohorts to simulate the treatment effects for both control and trial groups. The simulated cohort data not only can provide more information for patient recruitment and determination of the actual trial protocols, but also can increase the success rate and safety of the subsequent trial sessions.

Although we still lack the high-quality datasets and are unable to completely replace clinical trials, it is potential for us to employ drug clinical trials simulation to increase drug development efficiency (Kolla et al., 2021).

### 4.2 AI in optimizing drug treatment

Besides the applications related to clinical trials, AI can be used to optimize the therapeutic effects of drugs, which is important for clinical pharmacology. These applications include but are not limited to dosage of drug recommendations, individualized medical recommendations and effect prediction, adverse drug reactions, and prediction of drug-drug interactions (Johnson et al., 2023).

For drug dosage recommendations, both traditional machine learning (Vinks et al., 2020; van Gelder and Vinks, 2021; Bououda et al., 2022; Labriffe et al., 2022) and neural network (Yauney and Shah, 2018; Rödle et al., 2020) methods are widely used to estimate the amount of drugs. And then, we can optimize the efficacy of treatment while satisfying various constraints. For example, Rödle et al. (2020) developed an ANN model with backpropagation and genetic learning algorithm to predict the dosages of Ibuprofen, Paracetamol and Cefotaxime. The deviations of predicted dosage from real dosage of each medicine are 13%, 20% and 33%. As discussed by Rödle et al. (2020), it is urgent for this study area to have higher quality datasets, more indicators and outcome parameters to guarantee better development and application for drug dosage recommendation. Additionally, AI models are widely used in individualized treatment both in the static setting and time-dependent setting, including treatment recommendation, treatment outcome prediction, and individualized dose-response estimation. Potential data include patients’ personal information, electronic health records, diagnose data and so on. Detailed algorithms and methods of those studies are listed in the review by Bica et al. (2021).

Adverse drug reactions (ADR) (Martin et al., 2022) are also extremely important in the actual use of drugs, which means unexpected or unwanted effects caused by drugs. Improper use of drugs can lead to adverse reactions, causing additional illnesses or even deaths (Mohsen et al., 2021). Most of adverse drug reactions can be identified by toxicity-related predictions during pharmacokinetic parameter estimation (Basile et al., 2019), but some of them yet need to be predicted by AI models based on patients’ feedback and physiological data (Martin et al., 2022; Liu and Rudd, 2023). For example, Martin et al. (Martin et al., 2022) built up a predictive model for both ADR identification and seriousness assessment from structured and unstructured free-text information filled by patients, which employed TF-IDF + LGBM and Cross-lingual Language Model (XLM) to predict ADR identification. Here, XLM is an attention-based neural network and takes unstructured text data, while TF-IDF + LGBM takes additional structured data, like age, sex and so on. XLM and TF-IDF + LGBM both achieved an AUC of 0.97 on external validation, indicating the possibility to use of AI in the automatic pre-coding of pharmacovigilance reports. Meanwhile, the AI-based prediction and early detection of adverse drug reactions can effectively prevent the occurrence of adverse drug reactions and mitigate their consequences (Syrowatka et al., 2022).

Recently, it has become more and more common to adopt multi-drug combination therapy, but multiple drugs can easily inactivate some of them to affect the efficacy or even produce toxicity and cause additional complications. Therefore, the prediction of drug-drug interactions (DDIs) (Ryu et al., 2018; Zhang et al., 2023d) has become increasingly important.

With the increasing abundance of DDI-related databases, many machine learning and neural network models have been proposed to predict DDI and make great progress, which are detailed reviewed by Zhang et al. (2023d).

It is worth noting that the order of drugs administered may also affect the occurrence of DDIs, leading to asymmetric drug interactions. For example, a recent work by Feng et al. (2022) employs the directed graph attention network model DGAT-DDI to predict asymmetric drug interactions, in which source role encoder, target role encoder and self-role encoder are designed to represent how drugs influence and be influenced by other drugs and their chemical structures. Meanwhile, aggressiveness and impressionability are designed to capture the number of interaction partners and interaction tendencies. DGAT-DDI (Feng et al., 2022) achieved an AUC of 0.951, an AUPRC of 0.943 and an accuracy of 0.886 in the direction-specific task, and achieved an AUC of 0.867, an AUPRC of 0.854 and an accuracy of 0.795 in the direction-blind task. In the case study, seven of the top ten drug candidates in the model are validated by DrugBank, which demonstrates the practical capabilities of the model and the importance of further study on asymmetric drug interaction prediction (Feng et al., 2022; Zhang et al., 2023d).

## 5 Discussion and conclusion

AI have advanced many researches in biology (Zhang and Zhang, 2017; Zhang et al., 2018; Zhang et al., 2021e), disease (Li et al., 2017; Zhang et al., 2023a), cancer (Zhang et al., 2017a; Zhang et al., 2017b; Zhang et al., 2021b) and so do pharmacology. This review has briefly introduced the basic concepts of AI and the history of its development, and then summarized the applications of AI in pharmacology from three aspects: drug discovery and design, pharmacokinetic parameters estimation, and clinical pharmacology (Xia et al., 2017; Zhang et al., 2019a; Zhang et al., 2021a).

For these three aspects, we have listed relevant applications and major breakthroughs of AI in specific research fields, such as structure prediction, drug discovery, *de novo* drug design, clinical trial, and clinical drug therapy optimization. Although several research fields have not been mentioned, such as the application of AI in drug repositioning, drug manufacturing, and drug distribution, we listed them in the following articles (Paul et al., 2021; Tanoli et al., 2021; Yang et al., 2022).

It is noteworthy that AlphaFold has made great success in molecular structure prediction. Benefiting from the highly accurate prediction for 3D structures of a large number of molecules, it is easier for us to obtain structural information of targets in downstream drug discovery and design studies, thus providing the necessary prerequisite foundation for the discovery and design of novel drugs (Borkakoti and Thornton, 2023), and in turn, bringing a lot of new opportunities and ideas for drug discovery and design.

Therefore, it has become a widely discussed question: Can AI technologies and models, represented by AlphaFold, completely change the research of drug discovery and design?

Although most of the answers are “No”, we must note that AI research in drug discovery and design is constantly advancing and evolving, and AI models do not need to completely replace the research work of human. It’s a great advancement even if we just use AI models and tools to help accelerate the research for drug discovery and design, which will have great study potential in the distant future (Nussinov et al., 2023).

As discussed above, AI models have many advantages, including but not limited to the following: 1) AI models can perform more efficient calculations and predictions, and it can be demonstrated by ADMET online prediction tools such as AlphaFold and ADMETlab Version; 2) It is easier for us to employ deep learning language models to process unstructured text data than traditional machine learning, which is aforementioned by using XML to predict drug adverse reactions; 3) AI models have the potential to explore novel scientific knowledge and patterns, as evidenced by the graph-based attention network model DGAT-DDI mentioned above, which can be used to compute and explore asymmetric drug interactions.

However, there are still remaining many limitations and problems for AI models to solve. With the progress and success of AI models, how to collect data for AI model training becomes an increasingly important issue. The number of databases containing information on molecular structures, drug parameters, and drug interactions (Danishuddin et al., 2022; Sadybekov and Katritch, 2023; Zhang et al., 2023d) is fast increasing, which not only can provide a greater chemical space to explore new drugs, but also offer more data for better AI model training. However, it is worth noting that the large language model represented by ChatGPT and many other studies have pointed out that the quality of data is one of the most important factors in training an AI model, which suggests while expanding the amount of data, we should pay attention to the screening and quality control of the data (Aldoseri et al., 2023; Huang et al., 2023b; van der Lee and Swen, 2023; Whang et al., 2023). Moreover, with more and more databases available, the problems of overfitting, underfitting (Ying, 2019; Aliferis and Simon, 2024) and data imbalance (Krawczyk, 2016; Werner de Vargas et al., 2023) in AI deserve attention and vigilance. How to use some methods to avoid these problems as much as possible, such as cross-validation (Charilaou and Battat, 2022), regularization (Salehin and Kang, 2023), and data argumentation (Mumuni and Mumuni, 2022; Alomar et al., 2023), is also an important part of AI research.

Meanwhile, the interpretability of AI models deserves attention, though most of the current AI research does not take the model interpretability into consideration, such as face recognition or image processing (Zhang Q. et al., 2023). However, the interpretability of AI models has become a controversial issue for healthcare-related fields (Amann et al., 2020; Kırboğa et al., 2023). Many AI models are complex and lack explanations of the decision-making process causing these models to be termed as “Black-Box,” but explainable AI (XAI) models are trying to enhance transparency (Hassija et al., 2024). Research on XAI not only can alleviate people’s concerns about AI in drug research, but may also help medical and life science researchers discover the mechanisms and theories for the drugs and drug metabolism. Current research on XAI models has made great progress and has been applied in pharmacology related fields, but more exploration is still needed (Jiménez-Luna et al., 2020; Vo et al., 2022; Kırboğa et al., 2023; Hassija et al., 2024).

Also, the representation of molecules and drugs remains an important problem to be further discussed and studied. New algorithmic architecture that uses graph structure to represent molecules and employ graph neural networks to construct models has been wildly investigated with good progress, like ADMETlab, InteractionGraphNet, DGAT-DDI and many other methods mentioned before, but using graph structure to represent molecules still suffers predicament from insufficient expressive ability or too much complexity in some opinions (An et al., 2022). More practices and research are needed to explore the differences and applicable cases for both graph and traditional representation.

Despite the above problems and challenges, the applications of AI in pharmacology and medicine are still very valuable. As AI has made great success and breakthroughs in structure prediction, drug discovery and design, and pharmacokinetic parameter estimation, it is possible for us to build up an automated drug discovery and design platform by integrating these three research directions, a vision for future research. Moreover, it is foreseeable that AI models will gradually replace many previous traditional models, and even part of the work of humans. In this process, how to supervise, control and reasonably develop AI models will be an important issue to address and a future study direction.
